# NAD^+^ Promotes Superovulation of Huaxi Cattle Through Regulation of Cumulus Cell Proliferation and Oocyte Maturation

**DOI:** 10.3390/ijms26052276

**Published:** 2025-03-04

**Authors:** Song Wang, Mingcheng Liu, Anqi Di, Xiqing Jiang, Junjia Wu, Jiandong Zhang, Xuefei Liu, Chunling Bai, Guanghua Su, Lishuang Song, Guangpeng Li, Zhonghua Liu, Lei Yang

**Affiliations:** 1 Key Laboratory of Animal Cellular and Genetics Engineering of Heilongjiang Province, College of Life Science, Northeast Agricultural University, Harbin 150030, China; wangsong199852@163.com; 2 State Key Laboratory of Reproductive Regulation and Breeding of Grassland Livestock, College of Life Science, Inner Mongolia University, Hohhot 010070, China; 15047491247@163.com (M.L.); anqi_di@126.com (A.D.); jiangxq0903@163.com (X.J.); wujunjia0828@163.com (J.W.); m13150950609@163.com (J.Z.); liuxuefei1006@126.com (X.L.); chunling1980_0@163.com (C.B.); suguanghua0707@163.com (G.S.); xiaoshuang2000@126.com (L.S.); gpengli@imu.edu.cn (G.L.)

**Keywords:** superovulation, cumulus cells, oocytes, NAD^+^

## Abstract

Superovulation and embryo transfer are key technologies to improve the reproductive ability of female animals and enhance the efficiency of livestock production. However, poor-quality oocytes or abnormal fluctuations of hormone levels caused by superovulation affect the embryonic development environment, which may lead to a significant decline in the number and quality of transferable embryos, thus reducing the efficiency of superovulation. In this study, nicotinamide adenine dinucleotide (NAD^+^) was injected into Huaxi cows during the superovulation period to observe the proliferation and apoptosis of transplanted embryos. We examined the proliferation, apoptosis, reactive oxygen species (ROS) and mitochondrial membrane potential of cumulus cells and oocytes directly treated with NAD^+^ and investigated the potential mechanism of NAD^+^ to improve the superovulation efficiency by serum metabolomics and single-cell RNA sequencing. The results show that the addition of NAD^+^ significantly increased the quantity and quality of transferable embryos after superovulation. Differential metabolites during estrus synchronization and embryo flushing are enriched in glycerophospholipid metabolic pathways, suggesting that NAD^+^ can regulate lipid metabolic pathways. We found that NAD^+^ optimized the secretion levels of the steroid hormone estradiol (E2) and progesterone (P4) during superovulation by regulating the activity of cumulus cells. In oocytes, we found that NAD^+^ can inhibit apoptosis, scavenge ROS, and enhance mitochondrial function, thereby promoting oocyte maturation and enhancing embryo developmental potential. In conclusion, NAD^+^ significantly improved the superovulation ability of Huaxi cattle and provides an effective way for animal husbandry to improve the yield of high-quality embryos.

## 1. Introduction

Huaxi cattle is a new breed of beef cattle independently developed, with fast muscle growth, a high slaughter rate, superior meat production and excellent reproductive performance, all of which have reached the international advanced level [1,2]. In order to accelerate the expansion of the elite population of Huaxi cattle, superovulation technology has been used as an effective breeding method to improve the reproductive efficiency [3]. This technique makes it possible to rapidly expand the population by increasing the number of embryos [4]. However, the application of superovulation technology in Huaxi cattle is relatively limited, and poor oocyte quality or abnormal fluctuations in hormone levels may lead to a decline in quantity and quality of transferable embryos [5,6,7,8].

Nicotinamide adenine dinucleotide (NAD^+^) is a crucial factor involved in a variety of biological processes [9,10,11] and has important application potential in assisted reproduction and cell therapy [12,13,14,15]. Research has shown that NAD^+^ delays ovarian aging by improving mitochondrial function in adult zebrafish and mice [16,17]. NAD^+^ can also promote the secretion of estradiol (E2) and improve myocardial hypertrophy [18]. Previous studies have shown that NAD^+^ precursors, such as nicotinamide riboside (NR) or β-nicotinamide mononucleotide (NMN), can restore intracellular NAD^+^ levels, ameliorate mitochondrial dysfunction in cumulus cells, and promote blastocyst development [19,20,21,22], confirming the potential of NAD^+^ in improving embryonic development. However, the role and underlying mechanism of NAD^+^ in superovulation are unclear.

Therefore, this study investigated the effect of NAD^+^ on superovulation. Using metabolomic and transcriptomic analyses, we found that NAD^+^ could optimize the secretion of intraovarian steroid hormones by increasing the number of cumulus cells and directly modulate oocyte apoptosis and mitochondrial function, promoting oocyte maturation, and thereby improve superovulation efficiency. This study not only provides an important theoretical and practical basis for the application of NAD^+^ in bovine superovulation, but also instills new vitality and hope for the innovation of superovulation technology, the rapid growth of elite populations and the independent development of beef cattle breeding.

## 2. Results

### 2.1. NAD^+^ Promotes Superovulation in Huaxi Cattle

**Figure 1:**
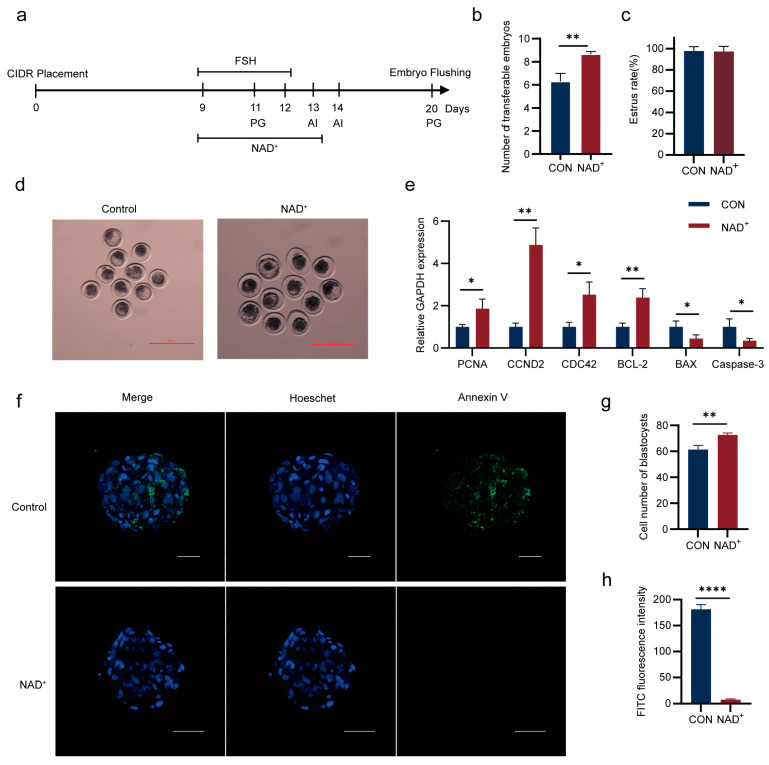
Nicotinamide adenine dinucleotide (NAD^+^) increased the quantity and quality of superovulatory transferable embryos in Huaxi cattle. (**a**) Schematic diagram of superovulation of Huaxi cattle; (**b**) The number of transferable embryos after NAD^+^ treatment. Histogram mean ± SD (N = 3), ** *p* < 0.01; (**c**) The estrus rate of Huaxi cattle after NAD^+^ treatment. The rate of estrus is the comparison of the number of superovulating cows that show an outgoing behavior with the total number of superovulated cows. Histogram mean ± SD (N = 3); (**d**) Representative photographs of recovered embryos with and without NAD^+^ treatment; (**e**) Relative expression levels of *PCNA*, *CCND2*, *CDC42*, *BCL-2*, *BAX* and *Caspase-3* mRNA in transferable embryos after NAD^+^ treatment. Histogram mean ± SD (N = 3), * *p* < 0.05, ** *p* < 0.01; (**f**) Representative confocal images of transferable embryos after NAD^+^ treatment. Green for Annexin V, blue for Hoechst. Scale, 100 μm; (**g**) The cell number in recovered blastocysts after NAD^+^ treatment. Histogram mean ± SD (N = 3), ** *p* < 0.01; (**h**) FITC fluorescence intensity of blastocysts after NAD^+^ treatment. Histogram mean ± SD (N = 3), **** *p* < 0.0001.

To test whether NAD^+^ enhances superovulation of Huaxi cattle, we injected 400 mg of NAD^+^ twice daily (morning and evening) from day 9 to day 13 of the superovulation period and evaluated the recovered embryos (Figure 1a). The results indicated that the number of transferable embryos in the NAD^+^ group was significantly higher than that in the control group (Figure 1b,d), while there was no significant difference in estrus rate (Figure 1c). The expressions of cell proliferation-related genes, Proliferating Cell Nuclear Antigen (*PCNA*), Cyclin D2 (*CCND2*), and Cell Division Cycle 42 (*CDC42*) and anti-apoptotic gene B-cell lymphoma-2 (*BCL-2*), were significantly increased in the NAD^+^ group. The expression levels of pro-apoptotic genes, Bcl-2-associated X (*BAX*) and Cysteinyl Aspartate Specific Proteinase-3 (*Caspase-3*), were significantly decreased (Figure 1e). Concurrently, the cell number in blastocysts in the NAD^+^ group significantly increased and the apoptosis decreased (Figure 1f–h), indicating that NAD^+^ improved the quality of transferable embryos.

### 2.2. NAD^+^ Promotes Lipid Metabolism During Superovulation in Huaxi Cattle

In order to further elucidate the molecular mechanism of NAD^+^ promoting superovulation in Huaxi cattle, metabolomic sequencing was performed on serum samples collected at different time points during the superovulation. According to the blood collection time, we divided the data into two groups for comparative analysis. Group I: Data collected on day 13; the control group was labeled as A, and the NAD^+^ group was labeled as B. Group II: Data collected on day 20; the control group was labeled as C, and the NAD^+^ group was labeled as D.

**Figure 2:**
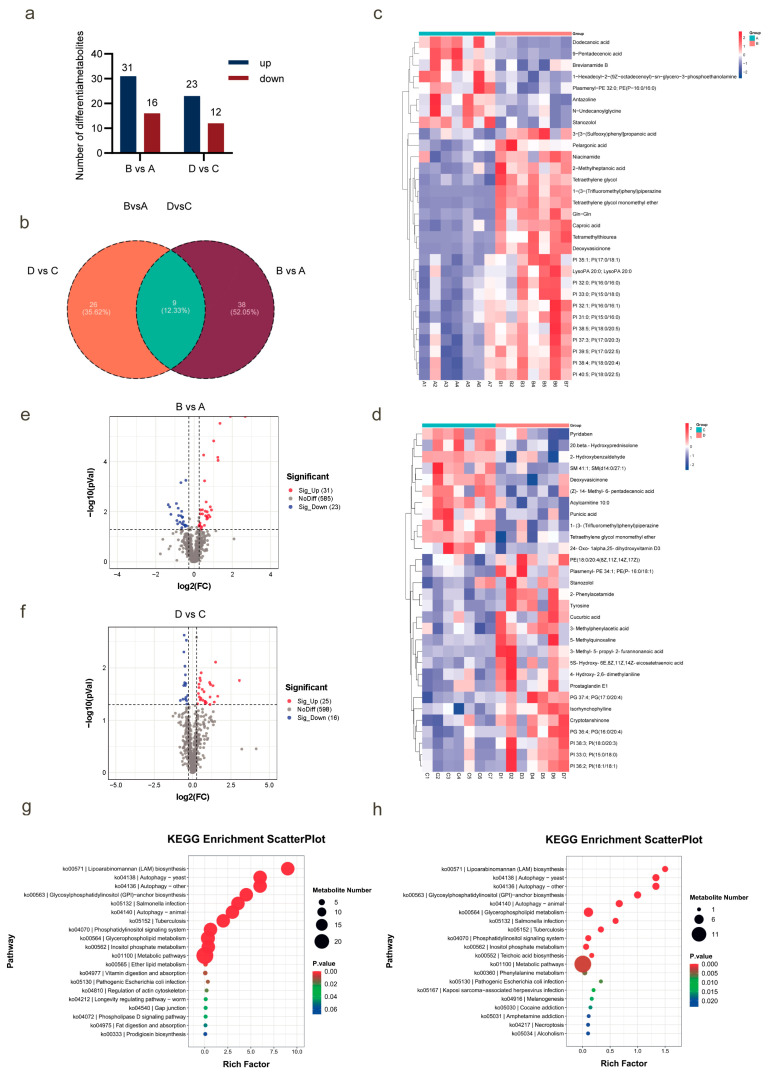
Metabolome sequencing at different time points during superovulation after NAD^+^ treatment. (**a**) Histogram of differential metabolites; (**b**) Venn diagram of differential metabolites; (**c**,**d**) Heatmaps of differential metabolites in Group I and Group II; (**e**,**f**) Volcanic maps of differential metabolites between Group I and Group II metabolites; (**g**,**h**) KEGG enrichment analysis of differential metabolites in Group I and Group II.

Based on the secondary metabolite annotation results from ion analysis, the differential metabolites were screened and counted. In Group I, a total of 47 differential metabolites were identified, of which 31 were upregulated and 16 downregulated. In Group II, a total of 35 differential metabolites were identified, of which 23 were upregulated and 12 downregulated (Figure 2a). To further explore the relationships among these differential metabolites, we used Venn diagram analysis to compare the differential secondary metabolites between different groups. The results show 9 differential metabolites in common between the two groups (Figure 2b). Among them, the metabolites in Group I were significantly upregulated, including nicotinamide, phosphatidylinositol and lysophosphatidic acid (*p* < 0.05), while the metabolites significantly downregulated were mainly phosphatidylethanolamine (*p* < 0.05, Figure 2c,e). The metabolites in Group II were significantly upregulated as phosphatidylinositol, tyrosine, 2-phenylacetamide and phosphatidylethanolamine (*p* < 0.05), and significantly downregulated as sphingomyelin (*p* < 0.05, Figure 2d,f).

Kyoto Encyclopedia of Genes and Genomes (KEGG) enrichment analysis revealed that several important metabolic pathways were identified in both Group I and Group II. In Group I, glycerophospholipid metabolism exhibited highly significant differences (*p* < 0.0001), and significant differences in ether lipid metabolism, vitamin digestion and absorption, and fat digestion and absorption (*p* < 0.05, Figure 2g). In Group II, glycerophospholipid metabolism also exhibited highly significant differences (*p* < 0.0001), and phenylalanine metabolism showed significant differences (*p* < 0.05, Figure 2h). Both Group I and Group II were enriched in the differential metabolite phosphatidylinositol and its associated glycerophospholipid metabolism pathways. These results indicate that NAD^+^ provides a more favorable environment for the growth and development of oocytes and embryos by regulating lipid metabolic pathways and thus significantly improves the superovulation efficiency of Huaxi cattle.

### 2.3. NAD^+^ Regulates Hormone Secretion During Superovulation in Huaxi Cattle

**Figure 3:**
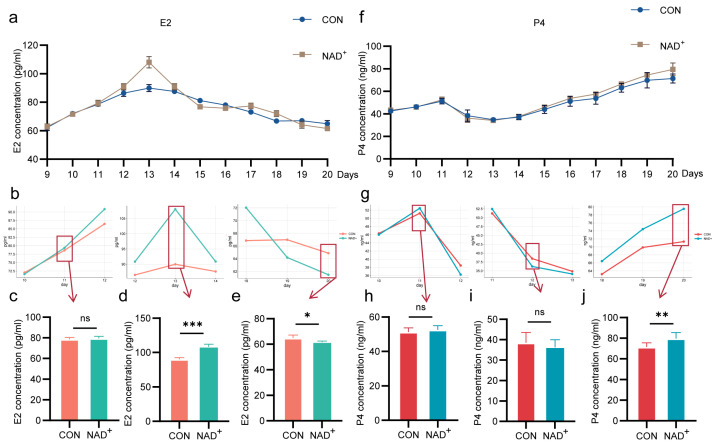
NAD^+^ regulated the concentrations of estradiol (E2) and progesterone (P4) during superovulation. (**a**) Serum E2 concentration after NAD^+^ treatment. Histogram mean ± SD (N = 3); (**b**) E2 concentration in partial time after NAD^+^ treatment; (**c**–**e**) E2 concentrations at day 11 (**c**), day 13 (**d**) and day 20 (**e**). Histogram mean ± SD (N = 3), * *p* < 0.05, *** *p* < 0.001, ns: not significant; (**f**) Serum P4 concentration after NAD^+^ treatment. Histogram mean ± SD (N = 3); (**g**) P4 concentration in partial time after NAD^+^ treatment; (**h**–**j**) P4 concentrations at day 11 (**h**), day 13 (**i**) and day 20 (**j**). Histogram mean ± SD (N = 3), ** *p* < 0.01, ns: not significant.

To investigate the effect of NAD^+^ on E2 hormone levels in the blood of the cows during superovulation, an enzyme-linked immunosorbent assay (ELISA) was used. The results show that the secretion trend of E2 in the NAD^+^ group was similar to that in the control group. At a specific time point, there were statistically significant differences in hormone concentrations between the two groups (*p* < 0.05, Figure 3a). Among them, from day 9 to day 13 of superovulation, E2 concentration in both the NAD^+^ group and the control group showed an increasing trend (Figure 3b,c). On day 13, E2 concentration in the NAD^+^ group was significantly higher than that in the control group (108.2 pg/mL vs. 90.0 pg/mL, *p* < 0.001, Figure 3b,d), and reached a peak at this time point. Then, from day 13 to day 20, the E2 concentration began to gradually decline. At day 20, E2 concentration decreased to the lowest level, and the NAD^+^ group was significantly lower than the control group (61.8 pg/mL vs. 64.9 pg/mL, *p* < 0.05, Figure 3b,e).

The serum progesterone (P4) concentration in both the NAD^+^ and control group was detected by ELISA. The results show that the secretion trend of P4 in NAD^+^ group was generally similar to that in the control group, exhibiting a fluctuating pattern of first increase, followed by a decrease, and then with second increase (Figure 3f). From day 9 to day 11, P4 concentration steadily increased (Figure 3g,h), reached the lowest point on day 13 (Figure 3g,i). Then, the P4 concentration began to rise again and reached the highest value on day 20. The concentration of P4 in the NAD^+^ group was significantly higher than that in the control group (79.6 ng/mL vs. 71.5 ng/mL, *p* < 0.01, Figure 3g,j).

The above results indicated that NAD^+^ significantly optimized the hormone secretion in Huaxi cattle, provided a more suitable internal environment for follicular growth, promoted the development of dominant follicles, and thus promoted oocyte maturation.

### 2.4. NAD^+^ Improves Hormone Synthesis by Promoting Proliferation of Cumulus Cells

**Figure 4:**
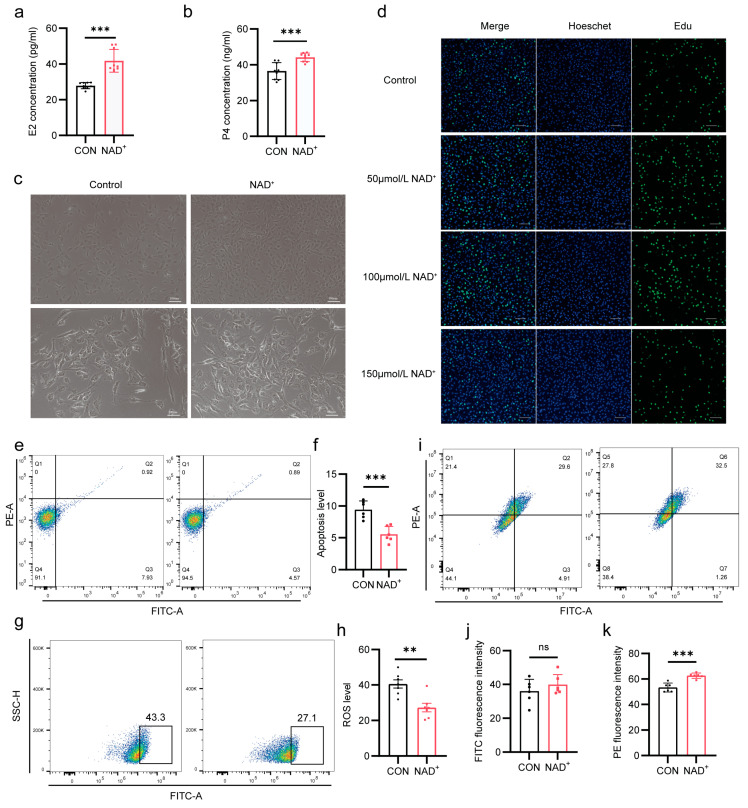
NAD^+^ promoted the proliferation and inhibited apoptosis of bovine cumulus cells. (**a**) E2 concentration in cumulus cells after NAD^+^ treatment. Histogram mean ± SD (N = 3), *** *p* < 0.001; (**b**) P4 concentration in cumulus cells after NAD^+^ treatment. Histogram mean ± SD (N = 3), *** *p* < 0.001; (**c**) Representative photographs of bovine cumulus cell growth. Upper scale, 100 μm. Lower scale, 200 μm; (**d**) Representative confocal images of cumulus cell proliferation after 24 h of NAD^+^ treatment. Edu is green, Hoechst is blue. Scale, 100 μm; (**e**,**f**) Representative flow cytometry (**e**) and statistical data (**f**) of cumulus cell apoptosis after NAD^+^ treatment. Histogram mean ± SD (N = 3), *** *p* < 0.001; (**g**,**h**) Representative flow cytometry (**g**) and statistical data (**h**) of ROS in cumulus cells treated with NAD^+^. Histogram mean ± SD (N = 3), ** *p* < 0.01; (**i**–**k**) Representative flow cytometry (**i**), FITC fluorescence statistics, ns: not significant (**j**) and PE fluorescence statistics (**k**) of mitochondrial membrane potential after NAD^+^ treatment. Histogram mean ± SD (N = 3), *** *p* < 0.001.

After digestion of the cumulus–oocyte complexes, the cumulus cells were collected. The cumulus cells were cultured in the medium supplemented with NAD^+^, and the supernatants were collected to detect the E2 and P4 concentrations by ELISA. The results show that the E2 and P4 concentrations were significantly increased after NAD^+^ treatment (*p* < 0.001, Figure 4a,b), indicating that NAD^+^ enhances the ability of cumulus cells to synthesize E2 and P4.

To further investigate whether NAD^+^ regulates the proliferation and development of cumulus cells and thus gain a deeper understanding of the potential mechanism by which NAD^+^ regulates superovulation, we observed that the cumulus cells showed stronger viability and a significant increase in cell number after NAD^+^ treatment (Figure 4c). An EdU fluorescence assay showed that the cell proliferation capacity was highest when NAD^+^ concentration was 100 µmol/L, indicating that NAD^+^ promoted the proliferation of cumulus cells (Figure 4d). However, this proliferation did not continue to increase with increasing NAD^+^ concentrations. Conversely, when the added NAD^+^ increased to 150 µmol/L, the proliferation capacity of cumulus cells decreased, suggesting that high concentrations of antioxidants may adversely affect the viability of cumulus cells. Therefore, in subsequent experiments, the concentration of NAD^+^ was set to 100 µmol/L. In addition, NAD^+^ reduced the apoptosis rate of cumulus cells from 7.93% to 4.57% (Figure 4e,f), indicating that NAD^+^ inhibited apoptosis of cumulus cells. Taking into account the antioxidant function of NAD^+^, we found that NAD^+^ significantly decreased reactive oxygen species (ROS) levels (*p* < 0.01, Figure 4g,h). NAD^+^ plays a regulatory role in cellular energy metabolism, so we further explored whether NAD^+^ affects mitochondrial function. Flow cytometry results show that mitochondrial membrane potential was significantly increased after NAD^+^ treatment (Figure 4i–k), indicating that NAD^+^ improved mitochondrial function. These results suggest that NAD^+^ not only directly promotes follicular development by enhancing hormone secretion of cumulus cells, but also forms a positive regulatory circuit by enhancing the anti-apoptotic capacity of cumulus cells, thus promoting follicular development and oocyte maturation.

### 2.5. NAD^+^ Directly Promotes Oocytes Maturation

**Figure 5:**
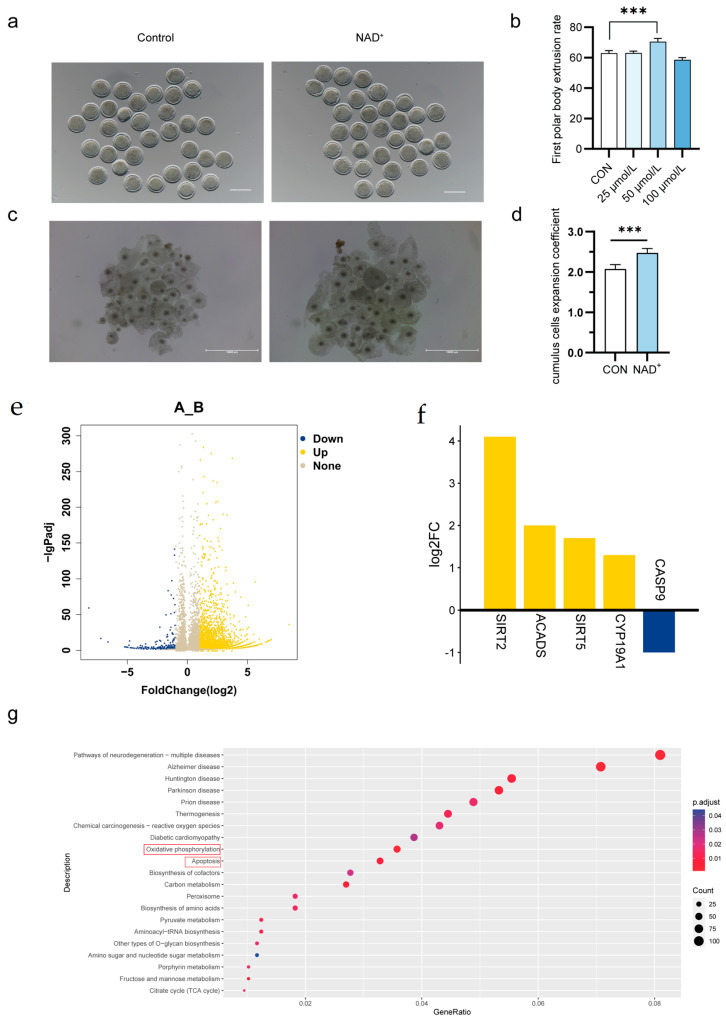
NAD^+^ promoted oocyte maturation and transcriptome analysis. (**a**) Representative photographs of mature oocytes treated with NAD^+^. Scale, 100 μm; (**b**) Oocyte maturation rate after treatment with different concentrations of NAD^+^. Histogram mean ± SD (N = 3), *** *p* < 0.001; (**c**) Representative photographs of cumulus cell expansion after NAD^+^ treatment after 24 h. Scale, 10,000 μm; (**d**) Expansion coefficient of NAD^+^-treated cumulus cells after 24 h of maturation. Histogram mean ± SD (N = 3), *** *p* < 0.001; (**e**) Volcanic maps of differentially expressed genes between control and NAD^+^ groups; (**f**) The differential gene expression levels between control and NAD^+^ group; (**g**) KEGG enrichment analysis of differentially expressed genes between control and NAD^+^ groups. Red boxes: pathways related to embryo development after NAD*+* treatment.

To further investigate the direct effect of NAD^+^ on oocyte maturation and embryonic development, non-superovulating bovine oocytes were cultured in vitro, and different concentrations of NAD^+^ (20 µmol/L, 50 µmol/L, 100 µmol/L) were added to the maturation medium. We assessed cytoplasmic maturity by measuring the expansion of cumulus cells 24 h after maturation. The nuclear maturation was verified by the extrusion of the first polar body. When 50 µmol/L of NAD^+^ was added, the first polar body extrusion rate of oocytes increased to 71.4% (Figure 5a,b). The expansion area of cumulus cells was larger and more uniform, and the expansion coefficient was significantly increased (Figure 5c,d). Therefore, in subsequent experiments, the concentration of NAD^+^ was set to 50 µmol/L.

To further investigate the effect of NAD^+^ on oocyte gene expression patterns, single-cell transcriptome sequencing was performed on oocytes in the NAD^+^ group and the control group. Compared with the control group, a total of 3401 differentially expressed genes were identified in the NAD^+^ group, including 3061 upregulated genes and 340 downregulated genes (Figure 5e). Among them, the *CYP19A1*, *ACADS*, *SIRT2*, and *SIRT5* genes were significantly upregulated, while the Caspase-9 gene was significantly downregulated (Figure 5f), suggesting that NAD^+^ may be related to biological events such as hormone secretion, mitochondrial function, cell metabolism, and apoptosis. KEGG pathway enrichment analysis showed that differentially expressed genes were mainly enriched in oxidative phosphorylation and apoptosis pathways, suggesting that NAD^+^ can promote energy metabolism and inhibit apoptosis, thus promoting oocyte development (Figure 5g).

**Figure 6:**
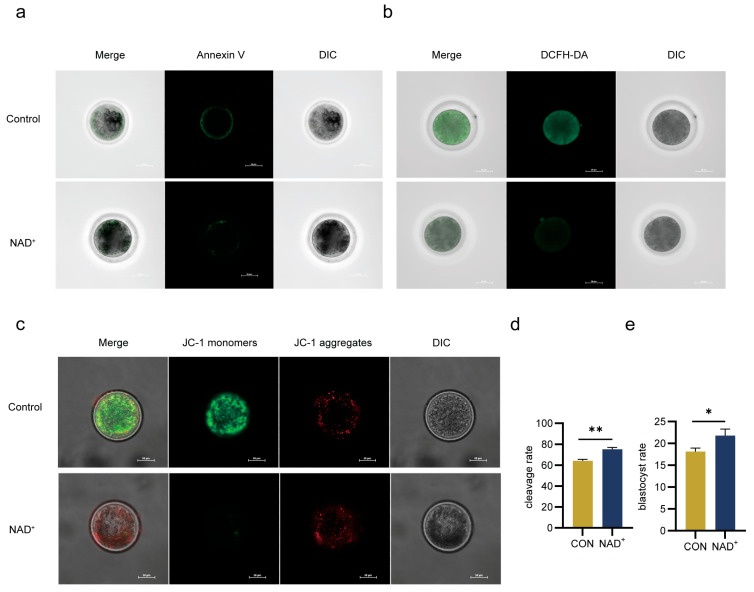
NAD^+^ inhibited oocyte apoptosis and ROS, improved mitochondrial function, and enhanced subsequent embryo development. (**a**) Representative confocal images of oocyte apoptosis after NAD^+^ treatment. Annexin V. Scale bar, 50 μm; (**b**) Representative confocal images of oocyte ROS after NAD^+^ treatment. Green for DCFH-DA. Scale, 50 μm; (**c**) Representative confocal images of mitochondrial membrane potential of oocytes treated with NAD^+^. JC-1 monomers in green and the JC-1 aggregates in red. Scale, 50 μm; (**d**) Cleavage rate after NAD^+^ treatment. Histogram mean ± SD (N = 3), ** *p* < 0.01; (**e**) Blastocyst rate after NAD^+^ treatment. Histogram mean ± SD (N = 3), * *p* < 0.05.

The addition of NAD^+^ significantly decreased oocyte apoptosis (Figure 6a) and ROS levels (Figure 6b) and significantly increased mitochondrial membrane potential (Figure 6c). The NAD^+^-treated oocytes were then subjected to in vitro fertilization, and the cleavage rate and blastocyst rate were significantly increased in the NAD^+^ group (Figure 6d,e). These results demonstrate that NAD^+^ improved oocyte quality by inhibiting apoptosis and ROS levels, enhanced mitochondrial function, and improved subsequent embryonic development.

## 3. Discussion

This study found that NAD^+^ could enhance the superovulation efficiency of Huaxi cattle. Metabolomic analysis indicate that NAD^+^ is involved in the regulation of lipid metabolism. Analyses of E2 and P4 indicated that NAD^+^ optimized the secretion of steroid hormones, promoted superovulation efficiency, and enhanced cumulus cell proliferation. In addition, transcriptome sequencing combined with subsequent validation experiments revealed that NAD^+^ can inhibit oocyte apoptosis, reduce ROS levels, improve mitochondrial function, promote oocyte maturation, and enhance subsequent embryonic development.

Cumulus cells play a central role in ensuring oocyte maturation and developmental potential. Specifically, they deliver key nutrients, including amino acids, nucleotides, and carbohydrate metabolites, to oocytes via gap junctions, thus laying a solid material foundation for oocyte maturation [23]. Regarding the regulation of cytoplasmic maturation of oocytes, cumulus cells participate in the metabolism of cystine. Specifically, cumulus cells can break down cystine into cysteine and promote its absorption by oocytes [24]. Interestingly, glutathione concentrations in bovine cumulus–oocyte complexes (COCs) were higher than in denuded oocytes [25]. The level of glutathione in oocytes is not only correlated with fertilization capacity of oocytes and the early development of embryos, but also plays a vital role in maintaining a cellular redox state and protecting cells from oxidative damage [26,27]. Moreover, the addition of cysteine to the culture medium can effectively increase the glutathione level in oocytes, thereby improving the efficacy of bovine in vitro maturation (IVM) [28]. Cumulus cells also influence oocyte maturation by changing intracellular pH or calcium ion (Ca^2+^) concentration [29]. Additionally, cumulus cells are capable of metabolizing glucose into pyruvate and then transferring it to oocytes, which helps to improve the quality of oocytes. In comparison with denuded oocytes, COCs have stronger metabolic capacity for glucose [30]. It is worth noting that when pyruvate is supplemented in the culture medium, bovine denuded oocytes can successfully complete the processes of maturation, fertilization and development [25].

E2 is a steroid hormone secreted by ovaries that promotes the maturation of developing follicles [31]. From superovulation to estrus induction, E2 concentration is negatively correlated with ovulation efficiency, while E2 concentration shows a positive correlation with ovulation efficiency during the estrus and breeding period. During embryo flushing, E2 concentration was again negatively correlated with ovulation efficiency [32]. P4 is a steroid hormone secreted by the corpus luteum and placenta that promotes follicular maturation and ovulation [31]. The blood P4 concentration was positively correlated with ovulation efficiency [32]. We found that injection of NAD^+^ during superovulation could optimize hormone secretion levels. This provides a solid foundation for subsequent sperm-oocyte binding and thereby ensures embryo development. Therefore, the role of NAD^+^ in optimizing hormone secretion levels is of great significance for improving the efficiency of superovulation.

NAD^+^ plays an active role in follicular development, oocyte maturation and embryo development [33]. However, reports on the regulation of cumulus cells by NAD^+^ are still limited. E2 is produced by cumulus cells, and P4 is secreted by the corpus luteum formed by cumulus cells and follicular wall cells [31]. It is worth noting that the concentrations of E2 and P4 in the follicles significantly regulate oocyte maturation [34]. Studies have shown that during the maturation of (COCs) in vitro, cumulus cells have the ability to secrete steroid hormones, thus playing a pivotal role in regulating oocyte meiosis [35,36,37]. In porcine oocytes, the rate of germinal vesicle breakdown has been shown to be closely related to the level of steroid hormones secreted by cumulus cells [38]. This clearly indicates that the maturation of oocytes is regulated by the level of steroid hormones secreted by cumulus cells. In the present study, we found that NAD^+^ promoted the proliferation of cumulus cells and increased the concentrations of E2 and P4 in cumulus cells. NAD^+^ reduced cumulus cell apoptosis, enhanced antioxidant capacity, and improved mitochondrial function. A close positive correlation existed between the state of cumulus cells and their hormone secretion. When cumulus cells were exposed to a higher concentration of hormones, their anti-apoptotic ability was significantly enhanced, thus maintaining a high level of cellular activity [39]. Based on the results of this study, we speculate that NAD^+^ not only directly promotes follicular development by promoting hormone secretion of cumulus cells, but also forms a positive regulatory circuit by enhancing the anti-apoptotic ability of cumulus cells, further promoting follicular development and oocyte maturation.

Oocytes obtain essential substances, such as amino acids, nucleotides, glutathione and sugars, through gap junctions with cumulus cells to maintain their energy requirements [39]. After NAD^+^ treatment, oocyte maturation rate was significantly increased and cumulus cell expansion was enhanced, which is consistent with the findings of Song et al. that addition of NMN improves porcine oocyte maturation [40]. RNA-sequencing analysis showed that *CYP19A1*, *ACADS*, *SIRT2* and *SIRT5* were significantly upregulated, while Caspase-9 was significantly downregulated. The enzyme encoded by *CYP19A1* is responsible for converting testosterone into estrogen, which in turn affects follicle survival and oocyte maturation [41]. During oocyte maturation, the upregulation of *CYP19A1* can also increase endogenous E2 levels, thus inducing autophagy and enhancing the early developmental potential of oocytes [41]. SIRT2 and SIRT5 are NAD^+^-dependent deacetylases that play important roles in energy intake, cell metabolism and mitochondrial function [42]. Studies have shown that the NAD^+^/SIRT2 pathway can prolong ovarian lifespan, delay oocyte senescence, and improve the quality of oocyte maturation [33]. In addition, unlike other family members, SIRT5 plays an important role in cellular antioxidant processes by inhibiting the activity of ACOX1, a metabolic enzyme that produces ROS, through desuccinylation, thereby reducing intracellular ROS levels [43]. Mitochondrial membrane potential and ROS stating were performed on oocytes in the NAD^+^ treatment group and the control group, which further confirmed that mitochondrial function of oocytes in the NAD^+^ treatment group was significantly enhanced, and the intracellular oxidative level was significantly reduced. Therefore, the upregulation of *SIRT2* and *SIRT5* genes is a key factor in improving the quality of oocyte maturation. Mitochondrial function is closely related to apoptosis because it involves Caspase-9, which can be cleaved and activated by cysteine proteases Caspase-3 and Caspase-7, thereby in turn cleaving numerous cellular substrates and leading to apoptosis [43,44]. Apoptosis staining showed that NAD^+^ enhanced the anti-apoptosis ability of bovine oocytes. The *ACADS* gene encodes short-chain acyl-CoA dehydrogenase (SCAD), and its upregulated expression can promote lipid catabolism. Genetic variation in *ACADS* leads to mitochondrial dysfunction, which in turn affects lipid metabolism [45]. Therefore, we found that during bovine oocyte maturation in vitro, NAD^+^ enhances lipid catabolism by upregulating mitochondrial activity, thus promoting fatty acid release and β-oxidation. This, in turn, increases the level of the intracellular energy substrate ATP, providing strong support for oocyte maturation quality.

Metabolomic sequencing showed that the differential metabolites in estrus included nicotinamide, phosphatidylinositol, lysophosphatidic acid, and phosphatidylethanolamine. During embryo flushing, differential metabolites contained phosphatidylinositol, tyrosine, 2-phenylacetamide, phosphatidylethanolamine and sphingomyelin. Phosphatidylinositol plays important roles in cell signaling, cell proliferation, differentiation and apoptosis [46,47]. During oocyte maturation and fertilization, various hormones and growth factors bind to their respective receptors and activate intracellular signaling pathways, thereby regulating oocyte meiosis, fertilization, and embryo development [48]. Phospholipid metabolism of oocytes and embryos is highly active, which is closely related to cell signaling and jointly determines cell fate and developmental process. Phosphatidylinositol promotes the synthesis of phosphatidylglycerol, which ultimately leads to the production of cardiolipin, the main phospholipid component of the inner mitochondrial membrane, which is essential for its characteristic structure. Cardiolipin is closely associated with the assembly and activity of various protein complexes in mitochondria, including respiratory chain complexes I to V and proteins in the solute carrier family, all of which have been shown to bind tightly to cardiolipin [49]. Lysophosphatidic acid (LPA) plays an important regulatory role in the field of reproduction and is involved in several crucial processes such as ovulation, fertilization, early embryo development, endometrial decidualization, angiogenesis and vascular remodeling. LPA is highly expressed in uterine epithelial cells during implantation and significantly decreased in patients with recurrent implantation failure [49]. Studies have shown that when sphingomyelin synthesis is highly activated, mouse uterine stromal cells can undergo decidualization under the regulation of progesterone. Blocking this synthetic pathway can significantly reduce defects in the implantation sites and decidualization process [50]. We speculate that NAD^+^ may be directly or indirectly involved in these biological processes, thereby enhancing cellular lipid metabolism during superovulation and providing necessary energy support for oocyte development and maturation. In addition, we found that in addition to lipid metabolites, differential metabolites also include amino acid metabolites. These metabolites are oxidized to aldehydes and ketones by enzymes in the peroxisomes. Among them, NAD^+^ acts as an electron transporter in the oxidative phosphorylation pathway, directly participates in the tricarboxylic acid cycle, and ultimately stores energy in the form of ATP, providing a sufficient energy source for oocyte maturation and embryonic development [51]. In summary, our findings indicate that NAD^+^ can not only optimize hormone secretion by promoting cumulus cell proliferation but also directly promote oocyte maturation, inhibit apoptosis, improve mitochondrial function, and reduce ROS production, thereby improving the efficiency of superovulation.

## 4. Materials and Methods

### 4.1. Ethics Statement

All experimental procedures used in this study were in accordance with the Regulation on the Administration of Laboratory Animals. All protocols were approved by the Animal Ethics Committee of Inner Mongolia University. This experiment was carried out with the approval of the ethics committee of experimental animals of Inner Mongolia University (No. IMU-CATTLE-2023-066).

### 4.2. Superovulation Treatment and Artificial Insemination

At day 0, a CIDR (Ningbo Sansheng Biotechnology Co., Ltd, Ningbo, China) containing 1.38g progesterone was placed into the vagina. FSH (Ningbo Sansheng Biotechnology Co., Ltd, Ningbo, China) was injected intramuscularly on day 9, and the dose of FSH gradually reduced over the next 3 days (Appendix A.1). On day 11, PG (Ningbo Sansheng Biotechnology Co., Ltd, Ningbo, China) was injected, respectively, in the morning and afternoon. On the morning of day 13, the cows were observed in estrus for 1 h. When the cows showed signs of estrus, insemination was conducted by transferring frozen–thawed semen into the uterine horns on both sides of the uterus on the evening of day 13. The second insemination was performed within 10 to 12 h. The semen used for insemination contained at least 35% motile spermatozoa. On day 20, the embryos were flushed and recovered. In the NAD^+^ group, 400 mg of NAD^+^ injection (100 mg/mL, Guangzhou Baiyunshan Pharmaceutical Co., Ltd., Guangzhou, China) was injected daily morning and evening from day 9 to day 13 following the designed superovulation protocol (Figure 1a).

### 4.3. Embryo Flushing

The cows were fixed, and caudal epidural anesthesia by 2% lidocaine (Ningbo Sansheng Biotechnology Co., Ltd, Ningbo, China) was performed. After cleaning and disinfecting the vagina area and the tails, the cervix was moderately dilated with a cervical dilater, and then the cervical mucus aspirator was inserted to extract the mucus. Then, the embryo tube with kernel was slowly introduced into the uterine horn; when the kernel reached the curved part of the uterine horn, it was moderately pulled out about 5 cm, and continued to push forward until it reached the front of the uterine horn at 1/3 to 1/2 position. Eight to 20 mL of air was filled into the embryo tube and connected the core of the embryo tube to the embryo tube. A 50 mL syringe was connected to the three-way catheter and clamped the output tube. The embryo flushing solution was then injected into the uterine horn through the input tube, and the input tube was clamped to allow the collected solution to flow into a 500 mL cell collector (FHK Fujihira Industry Co., Ltd., Tokyo, Japan) through the output tube. A syringe was used to aspirate 30 to 50 mL of embryo solution each time and rinsed repeatedly. The collected liquid was filtered in a cell collector at room temperature (18–25 °C), 10 mL of the liquid was retained, and the embryos were selected under a microscope (Nikon, Tokyo, Japan) at 20X magnification.

### 4.4. Serum Collection

Blood samples were collected into EDTA-containing serum-separator tubes. The blood samples were washed three times with PBS to remove plasma and white blood cells by centrifugation (1500 rpm/min, 15 min, 4 °C), and the accumulated red blood cells were collected. The sera from samples in serum-separator tubes were centrifuged (4000× *g*, 5 min, 4 °C), and supernatants were collected for analysis.

### 4.5. Evaluation of Oocyte Maturation After In Vitro Maturation (IVM)

Cumulus–oocyte complexes (COCs) were collected from slaughterhouse ovaries by aspirating the visible secondary follicles with a 10 mL syringe, and then the COCs were washed three times. The COCs were cultured in maturation medium (TCM199 with 10 % FBS, 10 IU/mL FSH, 10 IU/mL LH and 0.91 mM sodium pyruvate) for 24 h at a temperature of 38 °C in an incubator containing 5% CO_2_. After maturation, 0.1% hyaluronidase (Sigma, St. Louis, MO, USA) was added to COCs and vortexing was performed to remove cumulus cells. Then, oocytes with extruded first polar bodies were selected. We calculated the maturation rate, which was the percentage of oocytes releasing the first polar body among the total number of oocytes. The selected mature oocytes were transferred to the culture medium for subsequent experiments.

### 4.6. Culture of Cumulus Cells

The cumulus cells were collected by centrifugation of vortexed COCs at 1200 rpm for 5 min and washed three times with Dulbecco’s modified eagle medium (DMEM; Gibco, Grand Island, NE, USA) with 10% fetal bovine serum (FBS; 10099141, Gibco, USA), then cultured at 38 °C and 5% CO_2_.

### 4.7. RNA Extraction and Quantitative Real-Time PCR (qPCR)

We collected 20 embryos, extracted RNA using MiniBEST Universal RNA Extraction Kit (TaKaRa, Shiga, Japan), and performed Nanodrop analysis to determine sample concentration. The HiScriptII Q RT SuperMix for qPCR (Vazyme, Nanjing, China) was used to reverse-transcribe the extracted total RNA into cDNA. A ChamQ Universal SYBR qPCR Master Mix (Vazyme, Nanjing, China) kit and Roche LightCycler 480 real-time PCR (Roche, Basel, Switzerland) were used. Each experiment was repeated three times, and the gene expression level was calculated using R = 2^−[∆Ct sample − ∆Ct control]^. Primer information for qPCR is listed in Appendix A.2.

### 4.8. Apoptosis, ROS and Mitochondrial Membrane Potential Staining

Annexin V-FITC Apoptosis Detection Kit (Solarbio, Beijing, China) was used for apoptosis staining, the Reactive Oxygen Species Assay Kit (Solarbio, Beijing, China) was used for ROS detection, and the Mitochondria Membrane Potential Assay Kit with JC-1 (Solarbio, Beijing, China) was used to assay mitochondrial membrane potential. Images were captured with an AXR inverted laser confocal microscope (Nikon, Japan). Quantification analysis was carried out by ImageJ software 1.8.0. FACS analysis was performed using a Sony MA900 flow cytometer (Sony, Tokyo, Japan).

### 4.9. Sample Preparation and Analysis of Metabolomics

Serum metabolomics and analysis of NAD^+^ group and control were performed by Annoroad Gene Technology Co. LTD. A 100 μL sample was transferred to an EP tube and a 400 μL extraction solution (methanol: acetonitrile = 1:1, containing isotopic labeled in-ternal standard mixture) was added, vortexed and mixed for 30 s, sonicated for 10 min (ice water bath), left to stand at −40 °C for 1 h, then centrifuged at 12,000 rpm at 4 °C for 15 min. The supernatant was collected and subjected to ultra-high-performance liquid chromatography (Vanquish, Bangkok, Thailand). The target compound was isolated by Waters AC-QUITYUPLC BEH Amide liquid chromatography. Phase A of the liquid chromatography was aqueous, containing 25 mmol/L ammonium acetate and 25 mmol/L ammonia water, and Phase B was acetonitrile. Sample tray temperature: 4 °C, injection volume: 2 μL. The primary and secondary mass spectrometry data were collected by the Orbitrap Exploris 120 mass spectrometer. The raw data were converted into mzXML format by ProteoWizard software, processed by a self-developed R pro-gram package and matched with a secondary mass spectrometry database for substance annotation. The cutoff value for algorithm scoring was set to 0.3. XCMS software was used for peak extraction and quality control, and metaX software was used for metabolite identification. We annotated the identified metabolites by the commonly used functional databases and then conducted quantitative analysis of the metabolites. Differential metabolite function analyses, such as KEGG functional enrichment analysis, were performed.

### 4.10. Analysis of Single-Cell RNA-Seq

Single-cell RNA sequencing and analysis of oocytes from the control and NAD^+^ group were completed by Lianchuan Biotechnology Co., LTD. The FPKM (fragments per kilobases per million reads) method was used to normalize the mapped fragments. The DEGs between the control and NAD^+^ oocytes were identified by the DEG-seq software package by MA-plot-based random sampling (MARS) model. With a *p*-value < 0.05 and fold change absolute value > 2, the expression abundance was significant. All DEGs were mapped to terms in the KEGG database. The raw sequence data reported in this paper have been deposited in NCBI (ID: SUB15021884); open access is available at https://www.ncbi.nlm.nih.gov/geo/info/linking.html (accessed on 28 January 2025).

### 4.11. Statistical Analysis

All the data are expressed as mean ± SD. In the graphs, all the bars represent the means, while each error bar represents one standard deviation. When the standard deviations of the two groups were different, the two-tailed unpaired Student’s *t*-test was used for statistical analysis. * *p* < 0.05, ** *p* < 0.01, *** *p* < 0.001 and **** *p* < 0.0001 were considered to be statistically significant.

## Data Availability

The data generated and analyzed during this study are available upon reasonable request from the corresponding author.

## Appendix A

### Appendix A.1

**Table A1:** Time and dose of hormone injection.

Time	Day9		Day10		Day11		Day12		Day13		Day14		Day20
	AM	PM	AM	PM	AM	PM	AM	PM	AM	PM	AM	PM	
**Hormone**	FSH	FSH	FSH	FSH	FSH/PG	FSH/PG	FSH	FSH		AI	AI		PG
**Do** **se**	100	100	75	75	50/4	50/4	25	25					4
**(Unit)**	μg	μg	μg	μg	μg/mg	μg/mg	μg	μg					mg

### Appendix A.2

**Table A2:** qPCR primers used in this study.

Name	Primer Sequence (5′-3′)
PCNA	F: GTCCAGGGCTCCATCTTGAA
	R: CAAGGAGACATGAGACGAGT
CCND2	F: TGACCGCTGAGAAGTTATGC
	R: CGCCAGGTTCCATTTCAACT
CDC42	F: GTTGTTGTGGGTGATGGTGC
	R: TCCCCACCAATCATAACTGT
BCL-2	F: GAGTCGGATCGCAACTTGGA
	R: CTCTCGGCTGCTGCATTGT
BAX	F: GGCTGGACATTGGACTTCCTTC
	R: TGGTCACTGTCTGCCATGTGG
Caspase-3	F: TACTTGGGAAGGTGTGAGAAAACTAA
	R: AACCCGTCTCCCTTTATATTGCT
GAPDH	F: GGGTCATCATCTCTGCACCT
	R: GGTCATAAGTCCCTCCACGA
